# The Territorial Soldier and the Care of the Feet

**Published:** 1911-08-26

**Authors:** 


### The Royal Army Medical Corps Section.

### THE TERRITORIAL SOLDIER AND THE CARE OF THE FEET.

As the marching power of a regiment affects materially its usefulness on active service, it is very important that all the points influencing a high standard of efficiency in the walking power of the soldier should be carefully attended to. We do not propose to take up in detail all the hygienic rules that experience has laid down for observance by soldiers when on the march, but to confine our remarks to the care of the feet. When it is remembered that military statistics show that in a campaign, as a result of the first few days' marching, as many as 25 to 30 per cent. of the men suffer from some affection of the feet, and that it has been estimated that from this cause an average loss of 10 per cent. must be reckoned with amongst unseasoned troops when they first take the field, it is self-evident that, as regards the Territorial soldier, who may be suddenly called upon to perform field service, it is very necessary that the subject should have full consideration, and that not only regimental surgeons but also combatant officers, non-commissioned officers, and men should possess definite knowledge on it.

The three chief affections that cripple the marching soldier by giving rise to sore feet are: (1) Inflammation with swelling; (2) blisters; and (3) excoriations or abrasions. The first may be a localised condition, or it may involve the whole foot, which is red, hot, painful, and tender. Blisters may show themselves at any spot where there is great pressure and friction, so that they occur most commonly on the heel and at the heads of the metatarsal bones. In the same way the most painful and serious abrasions are usually situated on the heel at the level of the insertion of the tendo Achillis. Both blisters and abrasions cause great suffering to the soldier and disable him at once, so that remedial measures should be used immediately. In the case of swollen and inflamed feet the boots and socks should be removed as soon as possible and the feet washed and well dried. After this a clean pair of socks with easy shoes and elevation of the feet should soon put matters right. When blisters show elevation of the cuticle they should be emptied by inserting a clean needle into the sound skin about an eighth of an inch beyond the edge of the blister and passing it horizontally into the blister, when the fluid will run out beneath it. After evacuation the epidermal covering of the blister should be carefully flattened down and gentle pressure exercised on it by some lint or cotton wool, the latter being fixed in position by a strip of the strong American indiarubber plaster contained in the Army field pannier. Should the blister refill, the operation must be repeated, but this is usually not necessary. Experience has shown that the above is the best possible procedure to follow, as it allows the soldier to continue his march in comfort. Seeing that abrasions may easily pass by friction into a condition of ulceration, they should receive careful attention. After cleansing with an antiseptic lotion they should be dressed with some antiseptic ointment applied on lint larger than the sores and secured in position with strapping of strong plaster, or they should be covered with wadding soaked in a 10 per cent. solution of chromic acid. Under such management they should soon heal and allow the soldier to take his place again in the ranks.

Important, however, as is the treatment of the ailments of the soldier's feet, still more so is the prevention of them, and as the causes of them are not hard to find, there should not be insuperable difficulties in obviating them. The Territorial regimental surgeon may do a good deal by regular feet inspections in camp and by bringing before the officers and men of his unit the importance of the care of the feet, explaining to them how attention to what may seem minor details is of the very greatest assistance. One of the most common errors is neglect to keep the feet as clean as they ought to be. The value of frequent and thorough washing with soap and water should be insisted on. Where tenderness of the feet exists instruction should be given as to how this may be met by soaking the feet for a time in a solution of alum, salt, or saltpetre and water the night before a march, zinc or boric ointment being rubbed over them in the morning. If obtainable, a weak solution of formaldehyde of 1 to 200 is probably the best hardening solution for the feet, and excellent results have followed the plan of dusting into the socks the foot powder employed in the German army, composed of salicylic acid 3 parts, starch 10 parts, and powdered talcum 87 parts. In its absence finely pulverised alum dusted into the socks will be found a good substitute, as it diminishes the secretion and hardens the epidermis. Although the utility of the procedure has been called in question, there is a strong consensus of opinion that rubbing the socks inside and outside with soap or boracic ointment before marching is very useful.

While the enforcement of cleanliness and the adoption of the preventive measures just mentioned are of the greatest possible assistance, still more depends on the employment of good foot-gear, and the Territorial regimental surgeon should emphasise the great need of attention to this. He should point out the harm that accrues from unsuitable socks and ill-fitting boots. The socks should be free of any irritating dye and they should fit comfortably even after several washings. Above all they should not be too large, as then they are apt to crease and cause blisters. Socks, too, should be kept clean. When dirty they become hard and non-absorbent and loaded with germs. Any darning of a sock should be very carefully done so as not to leave an irregular surface. Probably the best sock for the Territorial soldier would be the seamless, ribbed, grey woollen sock of the Army pattern. So, too, in the matter of boots, it would be a good thing for the Territorial army if its members were all furnished with the "Colonial pattern" boot, which is in use in the Regular Army and has taken the place of the old ammunition boot. County Associations should move in this matter, as a good deal may be done by them. The existing arrangement under which the Territorial soldier provides his own boots and draws two shillings of boot money while in camp is not a good one. Some arrangement ought to be come to by which each Territorial soldier should be given on joining as a part of his kit a pair of Colonial pattern boots, and facilities should be given him for purchasing, at cost price, Army socks. Unfortunately, the military authorities have adopted the short-sighted policy of not allowing the County Associations to expend any money on boots or socks, so that nothing can be done in the matter of trying to improve the efficiency of the Territorial in this important detail. It is not too late, however, for wiser counsels to prevail and thus remedy what otherwise may be a source of inefficiency and a cause of weakness in our Army for Home Defence if called on for active service.

